# Updated distribution and new emerging populations of *Cynometracebuensis* F. Seid. (Fabaceae), a critically endangered endemic plant from the Philippines

**DOI:** 10.3897/BDJ.12.e132439

**Published:** 2024-10-01

**Authors:** Jeremaiah L. Estrada, Claudette I. Canonigo, Renerio P. Gentallan Jr.

**Affiliations:** 1 Department of Biology and Environmental Science, College of Science, University of the Philippines Cebu, Gorordo Avenue, Lahug, Cebu City 6000, Philippines Department of Biology and Environmental Science, College of Science, University of the Philippines Cebu Gorordo Avenue, Lahug, Cebu City 6000 Philippines; 2 Crop Breeding and Genetic Resources Laboratory, Institute of Crop Science, College of Agriculture and Food Science, University of the Philippines Los Baños, College 4031, Los Baños, Laguna, Philippines Crop Breeding and Genetic Resources Laboratory, Institute of Crop Science, College of Agriculture and Food Science, University of the Philippines Los Baños College 4031, Los Baños, Laguna Philippines

**Keywords:** *Cynometra*, Philippines, distribution, Cebu, endemic, critically endangered, forests over limestone

## Abstract

**Background:**

*Cynometracebuensis* F. Seid. is an endemic and threatened tree from the Philippines. The species was previously described to exclusively thrive in the Tabunan Forest of the Central Cebu Protected Landscape. Due to its narrow geographic distribution and threats from land conversion, slash-and-burn activities and non-regulated wood harvesting by locals, *C.cebuensis* was assessed as Critically Endangered (CR) under criteria B1a and B1b (as well as B2a and B2b) of the IUCN Red List in 2013. The present study provides new additional locations of *C.cebuensis* in the Municipalities of Argao and Minglanilla in Cebu along with population data and habitat characteristics for each site. Although new populations of *C.cebuensis* were discovered, it remains at significant risk of extinction in its natural habitat. Urgent and comprehensive conservation efforts are imperative to prevent its extinction.

**New information:**

This paper provides the updated distribution map of *Cynometracebuensis* F. Seid, a critically endangered endemic species in the Philippines showing five (5) distribution records.

## Introduction

*Cynometracebuensis* F. Seid, under the genus Fabaceae, is a critically endangered tree species endemic to Cebu Island, Philippines. Locally known as "nipot-nipot," it displays distinct single-branched bright pink tassels when young, which mature to a bright creamy greenish-yellow (Seidenschwarz 2013). This slow-growing species thrives in limestone forests at altitudes between 400 and 600 m above sea level (Seidenschwarz 2013). A decade after its discovery in the Tabunan Forest, the geographical range and distribution of *C.cebuensis* remain poorly understood and incomplete. This has led researchers to explore various areas across Cebu Island, particularly targeting dense forests and key biodiversity areas (KBAs).

The challenge in studying *C.cebuensis* arises from several factors: the absence of discernible flowers during field expeditions, the ephemeral nature of its blooms lasting only a few days in summer, its specific habitat preferences, and its low population density in forested areas. As a result, comprehensive reports on its biological and environmental characteristics are lacking, with only a limited number of published papers describing its morphology and distribution. Although some closely-related species with a cosmopolitan distribution are found in other Philippine regions (Pelser et al. 2011), this genus remains inadequately studied in terms of its distribution. Presently, there are only three published papers documenting its distribution, along with one available on a digital database featuring its photos and coordinates (Seidenschwarz 2013, Pelser and Barcelona 2017, Pelser and Barcelona 2018, Lillo et al. 2021, Lillo et al. 2023). To confirm if the species exclusively thrives in the Tabunan Forest and previously reported areas within Cebu Island, further exploration of additional areas is necessary to locate naturally occurring individuals of the species. Coordinates in widely accessible online digital databases are incomplete and no existing distribution map is available for this species. Therefore, this study aims to present updated distribution data of *C.cebuensis* within Cebu Island, Philippines, elucidating its geographical range and identifying its preferred habitats.

## Materials and methods

**Entry protocol and fieldwork**

Before conducting fieldwork and collecting samples in the forests of Cebu, Wildlife Gratuitous Permits No. 2024-09 and 2024-22 were obtained from the Department of Environment and Natural Resources Region 7, in accordance with the Philippine Republic Act No. 9147 (Wildlife Resources Conservation and Protection Act). The present study specifically conducted fieldwork in Mt. Lantoy in Argao, Cebu (latitude: 9.902500 and longitude: 123.529722) and from the Experimental Forest Station of DENR-CRERDEC in Minglanilla, Cebu (latitude: 10.325278 and longitude: 123.774444) from March to June 2024.

**Collection and taxonomic identification**

During the fieldwork, the species was primarily identified and collected with the help of forest guards and local guides. Leaves of the specimens were measured with a digital caliper and morphologically characterised, based on published studies by Seidenschwarz (2013) and Lillo et al. (2023). Co's Digital Flora of the Philippines (Pelser et al. 2011), a digital database of the native, naturalised, and invasive vascular plant species of the Philippines, was also used to verify species identity and confirm their presence in the areas. Samples were collected as voucher specimens and deposited in the Philippine Herbarium of Cultivated Plants at the Institute of Crop Science, University of the Philippines Los Baños. Photographs of *C.cebuensis* in its natural habitat were captured and processed using licensed Adobe Photoshop software to enhance visual clarity.

**Mapping the distribution of *C.cebuensis***

Location coordinates were recorded during sample collection and mapped using QGIS v. 3.36 Maidenhead. A thorough literature review was also conducted to collect and incorporate previously reported coordinates of *C.cebuensis* from published journals and online databases. All occurrence points collected for this study were incorporated into the updated distribution map.

## Taxon treatments

### Cynometra cebuensis

F. Seidenschwarz 2013

70D281D9-9BCE-5785-ADD7-3A2F68574C5B

https://www.gbif.org/species/8774275

https://www.ipni.org/n/77132497-1

https://tree.opentreeoflife.org/taxonomy/browse?id=7632457

https://powo.science.kew.org/taxon/urn:lsid:ipni.org:names:77132497-1

https://wfoplantlist.org/taxon/wfo-0001443714-2024-06?page=1

#### Materials

- **Type status:** Holotype. **Occurrence:** catalogNumber: CYNOC 20240615; occurrenceRemarks: Minglanilla, Cebu; recordedBy: Jeremaiah L. Estrada; individualCount: 17; lifeStage: wildling; reproductiveCondition: non-reproductive; establishmentMeans: endemic; occurrenceStatus: present; preparations: pressing/drying; disposition: critically endangered; occurrenceID: E6F92D2F-8E39-5E6F-BD96-F97FCBD76A92; **Taxon:** taxonID: Native; scientificNameID: https://powo.science.kew.org/taxon/urn:lsid:ipni.org:names:77132497-1#higher-classification; acceptedNameUsageID: https://powo.science.kew.org/taxon/urn:lsid:ipni.org:names:77132497-1#higher-classification; scientificName: *Cynometracebuensis*; acceptedNameUsage: *Cynometracebuensis* F. Seid; parentNameUsage: Fabaceae; originalNameUsage: *Cynometracebuensis* F. Seid; nameAccordingTo: Seidenschwarz F (2013) *Cynometracebuensis*, a new species of Leguminosae (Caesalpiniodeae) from the Philippines. Blumea 58: 19-20.; namePublishedIn: Seidenschwarz F (2013) *Cynometracebuensis*, a new species of Leguminosae (Caesalpiniodeae) from the Philippines. Blumea 58: 19-20.; higherClassification: Plantae; kingdom: Plantae; phylum: Streptophyta; class: Equisetopsida; order: Fabales; family: Fabaceae; genus: Cynometra; specificEpithet: *cebuensis*; taxonRank: species; verbatimTaxonRank: species; scientificNameAuthorship: Seidenschwarz; vernacularName: Nipot-nipot; nomenclaturalCode: ICN; taxonomicStatus: Accepted; taxonRemarks: Accepted name; **Location:** continent: Asia; island: Cebu; country: Philippines; stateProvince: Cebu; municipality: Minglanilla; locality: Camp 7, Minglanilla, Cebu, Philippines; verbatimElevation: 450; minimumElevationInMeters: 400; maximumElevationInMeters: 600; verbatimCoordinates: 10°19'35"N 123°46'26"E; verbatimLatitude: 10°19'35"N; verbatimLongitude: 123°46'26"E; verbatimCoordinateSystem: Degrees, minutes, seconds,; decimalLatitude: 10.3243; decimalLongitude: 123.7755; georeferenceProtocol: Georeferencing Quick Reference Guide (Zermoglio et al. 2020, https://doi.org/10.35035/e09p-h128); **Identification:** identificationID: Cynometracebuensis; identifiedBy: Jeremaiah L. Estrada; dateIdentified: 2024-06-15, 10:10 AM; identificationReferences: Seidenschwarz F (2013) Cynometracebuensis, a new species of Leguminosae (Caesalpiniodeae) from the Philippines. Blumea 58: 19-20.; **Event:** eventID: \; samplingProtocol: Observation; samplingEffort: 3 observer hours; eventDate: 06-15-24; eventTime: 10:00 AM; startDayOfYear: 01-01-24; endDayOfYear: 12-31-24; year: 2024; month: 6; day: 15; verbatimEventDate: 15-Jun-24; habitat: Dry forest over limestone; eventRemarks: The species grows in dry forest over limestone. Several wildlings were identified.; **Record Level:** type: Living plant; language: en; rightsHolder: Jeremaiah L. Estrada; institutionID: UPLB Philippine Herbarium of Cultivated Plants; collectionID: Estrada and Canonigo 004; institutionCode: UPLB; collectionCode: Plant; ownerInstitutionCode: UPLB; basisOfRecord: PreservedSpecimen

#### Description

**Figure 1.**
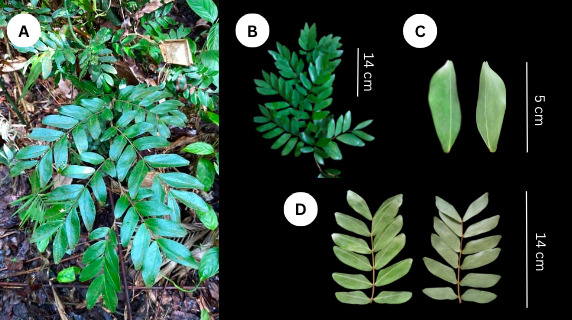
*Cynometracebuensis* wildling from the forests of Argao and Minglanilla, Cebu (A) in its natural habitat showing its branch (B) and leaflets (adaxial, abaxial) (C-D).

**Morphology**: The species is a treelet reaching up to approximately 12 m in height with no buttresses. The leaves are composed of (3–)4–6 leaflets arranged in pairs; the petioles are rugose, measuring 5–7 mm in length and both the rachis and petioles are covered in hairs. The leaflets are attached directly to the stem, dark green and have an ovate to oblong form, measuring between 2–5(–6) cm in length and 0.8–1.5(–2.2) cm in width. The leaflets feature a deeply notched tip and an asymmetrical base, with the acroscopic side being wedge-shaped or cuneate and the basiscopic side rounded or auriculate. The midrib is positioned 5–7 mm from the acroscopic edge and gives rise to 7–9 pairs of lateral veins. The margin of the leaflets is thickened (Seidenschwarz 2013, Lillo et al. 2023) (Fig. 1).

#### Distribution

**Figure 2.**
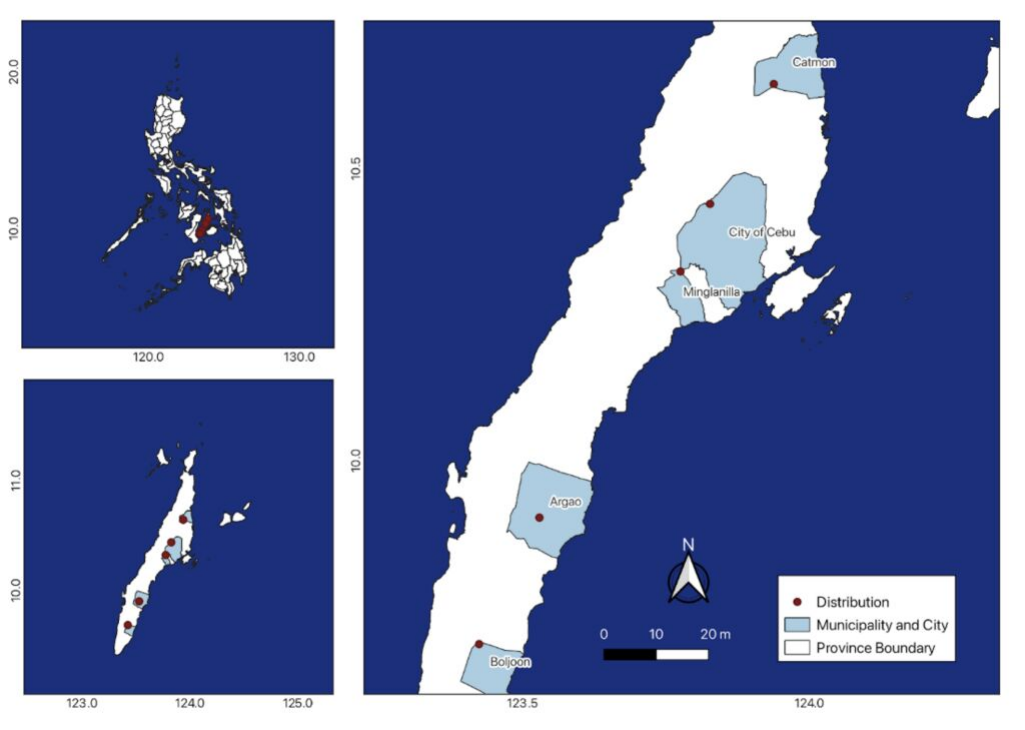
The updated distribution map of *Cynometracebuensis* in the Philippines with a new distribution record.

Cebu Island, Philippines. Tabunan Forest, Cebu City; Mt. Kapayas, Carmen, Cebu; Boljoon, Cebu; Mt. Lantoy, Argao, Cebu; Camp 7, Minglanilla, Cebu* (new record) (Table 1; Fig. 2).

#### Ecology

**Figure 3.**
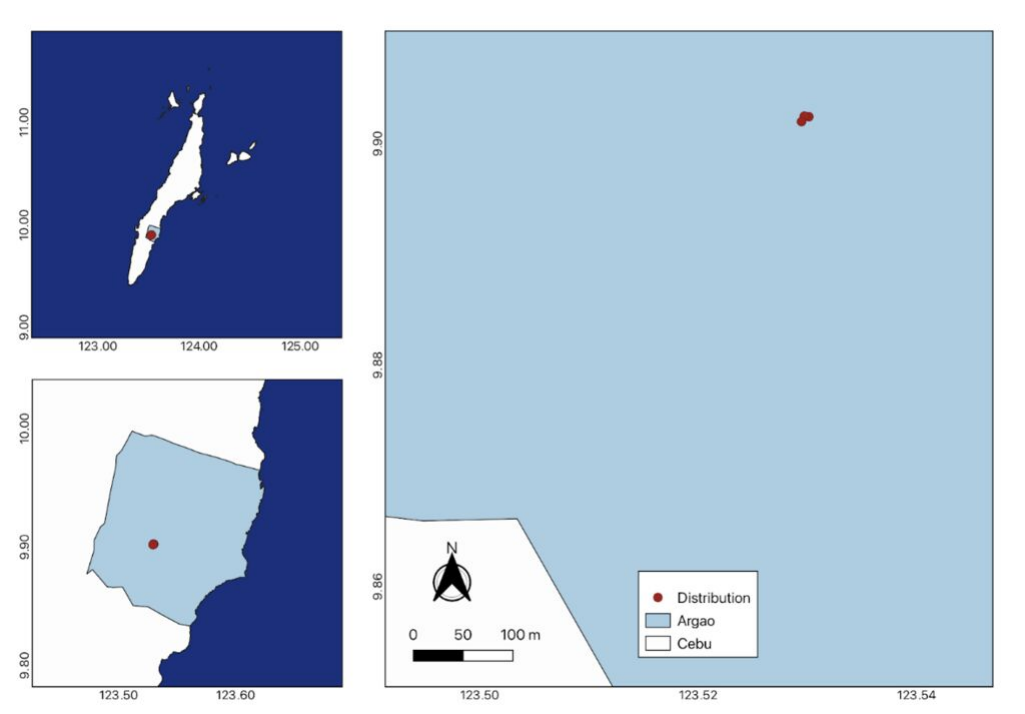
Population of *Cynometracebuensis* in Argao, Cebu.

**Figure 4.**
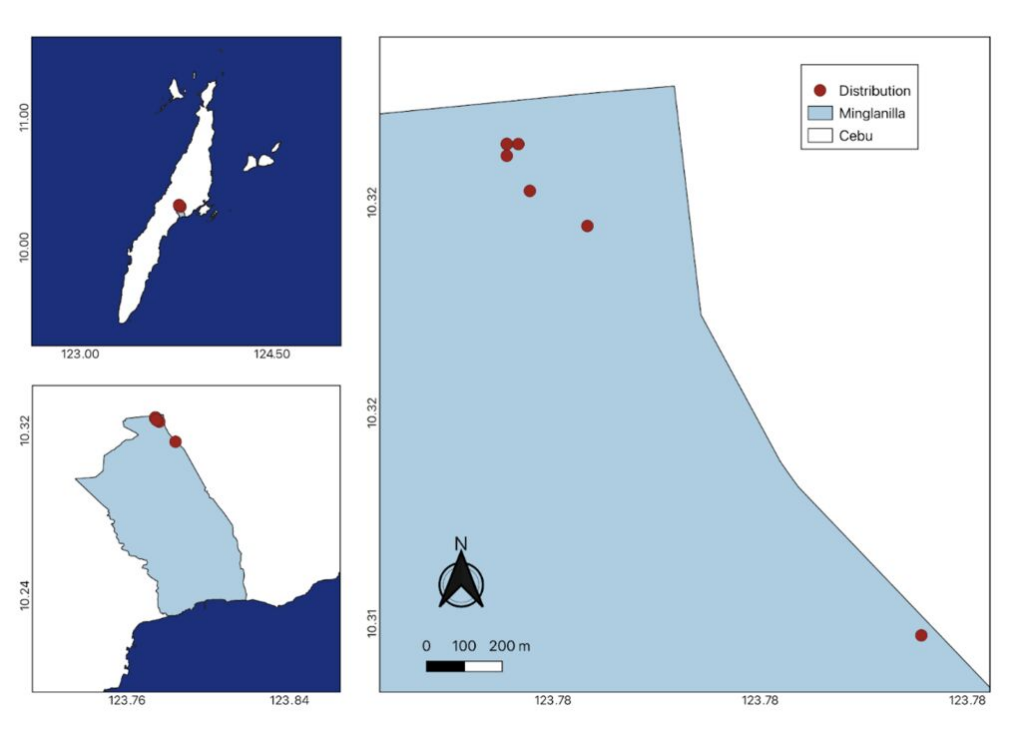
New emerging population of *Cynometracebuensis* in Camp 7, Minglanilla, Cebu.

In contrast to its closely-related species, such as *Cynometracopelandii* and *Cynometrawarbugyii*, which thrive near waterbodies (Seidenschwarz 2013, Lillo et al. 2023), this species specifically flourishes in dry forests over limestone terrain at elevations of 400 m above sea level or higher. For the phenology of the species, flowering season occurs during the onset of the dry season, from March to April. Flowers are reported to last only a few days, which may be due to inadequate or rapid pollination, environmental conditions and photoperiodism. Its fruits are reported to develop within 3 months and fall from the trees in July (Seidenschwarz 2013). Wildlings were discovered thriving in shaded areas of the forest, alongside other forest species. New populations of *C.cebuensis* were discovered in Mt. Lantoy, Argao, Cebu (Fig. 3); however, a larger population was found in Camp 7, Minglanilla, Cebu. Eighteen (18) wildlings were identified in Minglanilla during fieldwork in June 2024 (Fig. 4).

#### Conservation

*Cynometracebuensis* was previously assessed for the IUCN Red List of Threatened Species in 2021. It was listed as Critically Endangered under criteria B1ab(iii) and 2ab(iii) due to its restricted geographic range, limited occurrence and habitat degradation brought about by non-regulated timber harvesting, land conversion and urbanisation. The forests over limestone where the species grows are surrounded by residential and agricultural areas, making its population highly vulnerable to anthropogenic activities and fires during the dry months (March to April).

#### Biology

*C.cebuensis* is known for its slow growth rate. Flowers typically develop nine years after planting *in situ*. After ten years, the species reaches an average height of up to 8 m and an average stem diameter of 9 cm. Additionally, it produces distinct pink, single-branched tassels several times a year (Seidenschwarz 2013).

#### Notes

No fertile specimens were observed or collected during the study period in Argao and Minglanilla, Cebu. Voucher specimens were deposited at the Philippine Herbarium for Cultivated Plants at the Institute of Crop Science, College of Agriculture and Food Science, University of the Philippines Los Baños.

## Discussion

*Cynometracebuensis* was initially discovered and described in Tabunan Forest, located in the Central Cebu Protected Landscape on Cebu Island, Philippines (Seidenschwarz 2013, Lillo et al. 2023). Subsequently, three years later, Pelser and Barcelona (2017) documented the species in Brgy. Cansuje, Argao and in the Municipality of Boljoon, Cebu. This observation was further supported by Lillo et al. (2023), who reported the species' occurrence in Mt. Lantoy in Argao, along with another distribution record in Mt. Kapayas in Carmen, Cebu (Lillo et al. 2021). These reported occurrence points were not integrated into a single map nor submitted to the Global Biodiversity Information Facility (GBIF). In this study, specimens of *C.cebuensis* were observed in limestone forests at both Mt. Lantoy in Argao and Camp 7 in Minglanilla, Cebu. The species' presence in Minglanilla marks a new distribution record. In addition to previously published data, it can be inferred that the species predominantly inhabits dry forests situated over limestone at an altitude of 400 m above sea level. Notably, younger and smaller individuals, with a maximum height of 3 m, were identified in Mt. Lantoy, while taller counterparts, reaching up to 5 m, were found in Minglanilla, Cebu. Only three wildlings were found on the forest floor of Mt. Lantoy, whereas 18 wildlings were discovered in Minglanilla, Cebu.

The updated distribution map of *C.cebuensis* generated in this study holds important implications for understanding the suitable habitats of the critically endangered species, providing insights into its current geographical range and distribution patterns. Findings from this study refute the previously held restrictions about the endemicity of *C.cebuensis* in Tabunan Forest (Seidenschwarz 2013) indicating its wide distribution range and resilience across sites (Lillo et al. 2023). However, it is important to note that the species grows along trails and in nearby residential areas, which can impact its population growth due to the unregulated harvesting of wood from the forests. The medicinal and pharmaceutical potential of the plant also remains largely untapped due to its rarity and slow growth rate. Accessing large amounts of leaves for pharmacological screening is restricted by Department of Environment and Natural Resources (DENR) regulations, as the plant is critically endangered. Acquiring a Wildlife Gratuitous Permit from DENR is necessary for this purpose. As per Seidenschwarz (2013), the species is characterised by slow growth, typically reaching an average height of 8 m with a stem diameter of 8.6 cm after 10 years. The general public is often unaware of their slow growth rate, which can be detrimental to the species. According to a staff member from DENR-Community Environment and Natural Resources Office in Argao, these trees are often cut down for fencing and firewood before they reach maturity. In terms of its phenology, it only begins flowering nine years after planting, making the collection of seeds and fruits challenging (Seidenschwarz 2013). Identifying the species quickly during fieldwork is also difficult due to this factor. We suggest collecting seeds from the species to establish plant sanctuaries to support local pollination and exploring methods such as *in vitro* micropropagation for its cultivation. We also recommend exploring uncharted areas within Cebu Island to find the species and use ecological niche modelling to predict its distribution, taking into account climate data and the edaphic factors of its environment. By doing so, the current population and its resilience will be assessed, allowing for a re-assessment of its conservation status using the Red List Index (RLI).

The discovery of *C.cebuensis* in additional locations, especially in key biodiversity areas (KBAs), underscores the significance of these sites in terms of habitat suitability. This suggests that these locations offer optimal environmental conditions for the species' growth and may serve as crucial habitats, highlighting the urgency of their protection and conservation. The collective effort, support and collaborations with various stakeholders such as local government units (LGUs), national government agencies (NGAs), academic and research institutions and non-governmental organisations (NGOs) are essential for the protection of our critically endangered and endemic species like *C.cebuensis*.

## Supplementary Material

XML Treatment for Cynometra cebuensis

## Figures and Tables

**Table 1.** Distribution and coordinates of *Cynometracebuensis* F. Seid. in the different municipalities of Cebu Island, Philippines.

**Location**	**Latitude**	**Longitude**	**References**
Tabunan Forest, Cebu City	10.44005	123.8271	Seidenschwarz (2013)
Brgy. Cansuje/Mt. Lantoy, Argao, Cebu	9.9025	123.5297	Pelser and Barcelona (2017), Lillo et al. (2023)
Boljoon, Cebu	9.685556	123.4246	Pelser and Barcelona (2018)
Mt. Kapayas, Carmen, Cebu	10.64531	123.9381	Lillo et al. (2021)
Camp 7, Minglanilla, Cebu	10.3243	123.7756	This study
